# Automatic 4D Reconstruction of Patient-Specific Cardiac Mesh with 1-to-1 Vertex Correspondence from Segmented Contours Lines

**DOI:** 10.1371/journal.pone.0093747

**Published:** 2014-04-17

**Authors:** Chi Wan Lim, Yi Su, Si Yong Yeo, Gillian Maria Ng, Vinh Tan Nguyen, Liang Zhong, Ru San Tan, Kian Keong Poh, Ping Chai

**Affiliations:** 1 Institute of High Performance Computing, A*STAR, Singapore, Singapore; 2 National Heart Centre Singapore, Singapore; 3 Cardiac Department, National University Heart Center, National University Health System, Singapore, Singapore; 4 Department of Medicine, Yong Loo Lin School of Medicine, National University of Singapore, Singapore; University of Minnesota, United States of America

## Abstract

We propose an automatic algorithm for the reconstruction of patient-specific cardiac mesh models with 1-to-1 vertex correspondence. In this framework, a series of 3D meshes depicting the endocardial surface of the heart at each time step is constructed, based on a set of border delineated magnetic resonance imaging (MRI) data of the whole cardiac cycle. The key contribution in this work involves a novel reconstruction technique to generate a 4D (i.e., spatial–temporal) model of the heart with 1-to-1 vertex mapping throughout the time frames. The reconstructed 3D model from the first time step is used as a base template model and then deformed to fit the segmented contours from the subsequent time steps. A method to determine a tree-based connectivity relationship is proposed to ensure robust mapping during mesh deformation. The novel feature is the ability to handle intra- and inter-frame 2D topology changes of the contours, which manifests as a series of merging and splitting of contours when the images are viewed either in a spatial or temporal sequence. Our algorithm has been tested on five acquisitions of cardiac MRI and can successfully reconstruct the full 4D heart model in around 30 minutes per subject. The generated 4D heart model conforms very well with the input segmented contours and the mesh element shape is of reasonably good quality. The work is important in the support of downstream computational simulation activities.

## Introduction

Cardiovascular diseases are becoming more commonplace in modern societies. Computed Tomography (CT) and Magnetic Resonance Imaging (MRI) are both non-invasive techniques that are often used by physicians to view the internal cross-sectional images of a patient's heart with cardiovascular disease. Using computational methods in cardiac analysis can allow us to further understand and visualize what we are unable to obtain from static 2D images, such as blood flow behavior (hemodynamics) in the chambers. To achieve this, the reconstruction of the 4D (spatial temporal) model of the heart is an important and critical requirement. Many MRI-based 4D heart reconstruction studies have focused primarily on only the left ventricle (LV), in part due to the relatively intricate configuration of the pulmonary veins and right atrium. In this paper, we propose a framework to directly reconstruct a 4D left heart model (i.e., from the pulmonary veins to the LV apex) from segmented contours drawn on MRI images. Our proposed framework has the following advantages:

- The algorithm does not require any form of landmark placement.
- The 4D model has 1-to-1 vertex correspondence across all the frames of the cardiac cycle.
- 2D contour topological changes are handled automatically in both the spatial and temporal dimensions.

Four-dimensional reconstruction of the heart would be useful for the visualization of complex geometries of the heart chambers, especially prior to surgical intervention. For instance, in patients with left ventricular aneurysms, the ability to visualize the aneurysm and its relation to the left ventricular myocardium would be helpful for surgical planning before aneurysmectomy. Full 4D modelling of the left heart in cases of valvular heart disease may allow physicians to understand more about the cardiac remodelling process and evaluate the effects of therapeutic intervention on the disease process. Other disease conditions in which 4D modelling may potentially be useful for clinical decision making include congenital heart diseases, cardiac/paracardiac tumours and right heart pathologies affecting the left heart.

### Related work

Cardiac reconstruction, either static (3D) or dynamic (4D), has traditionally focused on the ventricular regions due to their simpler morphology and the relative ease of border delineation on CT and MRI scans, as compared to the atrial regions. This differentiation is further compounded for MRI-based methods due to its relatively large spatial intervals between slices. A comprehensive review of the different modeling techniques from cardiac images prior to 2001 was presented by Frangi *et al.* [1].

One of the first studies to directly construct a 3D model of the heart is by McQueen and Peskin [2], which employed idealized cones and ellipsoids for modeling the left and right ventricles for simulating cardiac hemodynamics [3]. Using 3D echocardiography, Corsi *et al.* [4] employed the level set method and marching cubes algorithm to reconstruct a 3D model of the LV. Zhukov *et al.* [5] proposed to deform a sphere model using dynamic remeshing and curvature estimation methods to produce high quality meshes of the heart. Montagnat and Delingette [6] extended the deformable surface framework by introducing time-dependent constraints such as temporal smoothing and trajectory constraints. Sermesant *et al.* [7] fitted images from various modalities together using a non-rigid registration approach to create a mesh model of both the LV and RV (right ventricle) using the GHS3D commercial software. Using segmented 3D MR images, Škrinjar and Bistoquet [8] mapped a premeshed sphere to the surface of the segmented volume to generate the surface meshes of the epicardium and endocardium of the four cardiac chambers. What makes the construction of a MR-based patient-specific cardiac model that connects the atria and the connecting arteries to the ventricles particularly challenging is the strongly anisotropic voxels in the long-axis direction. A noteworthy attempt to reconstruct a hugely detailed static animal heart model is presented by Plank *et al.* [9], which uses a 9.4T MR system that is able to generate MR datasets with isotropic resolution of up to 20 µm.

Tagged MRI is a popular imaging modality for 4D cardiac reconstruction due to its capability in capturing myocardium motion, especially for the ventricles. Lou and Heng [10] modeled the LV as a generalized prolate spheriod, and using B-splines to model the LV motion in terms of translation, polar radial compression, twisting and bending from tagged MR images. Wang *et al.* [11] employed a generic finite element model adapted from L*ö*tj*ö*nen *et al.* [12] and deformed it using image forces computed from the tagged MRI to model the motion of the LV. Using the same FE model, Zhang *et al.* [13] applied laplacian surface deformation to construct the surface of the LV by deforming from a generic model, and subsequently used a meshless deformable approach [14] to avoid the constant need for remeshing irregular regions. In the work by Park *et al.* [15], myocardium motion extracted from tagged MR was used to deform a generic finite element model by using a latitude-longitude parameterizing approach. Schenkel *et al.* [16] proposed a block structured hexahedral type grid representation for each segmented contour in order to reconstruct a LV model with a structured mesh for performing computational fluid dynamics flow analysis. However, the usage of tagged MRI to aid in the deformation process is mostly restricted to the ventricles, as the large inter-slice distance combined with significant distortion occurring near the valve regions contribute to the difficulty of resolving the motion displacement at the atria.

According to Frangi *et al.* [1], incorporating prior shape knowledge in the segmentation process is advantageous as it allows segmentation, image analysis and shape modeling to be combined into a single framework. A recent review of segmentation methods for short-axis cardiac MR images was presented by Petitjean and Dacher [17]. One of the approaches is to use statistical shape modeling by training on a set of sample models [18] to obtain a set of variability parameters. L*ö*tj*ö*nen *et al.* [19] made use of both long- and short-axis MR images to create such a statistical model of the atria, ventricles and epicardium using a point distribution model, but these model components are disjoint from each other. Berg and Lorenz [20] went on to create a full heart model by using generic shapes such as spheres (atria), tubes (vessels), and ellipsoids (ventricles) to model the main cardiac anatomies. However, it requires manual placement of landmarks for initialization. Ecabert *et al.* [21] improved on the robustness of the technique by including *a priori* shape knowledge, and later using a 3D implementation of the generalized Hough transform [22] for better initial localization of the image. Zhang *et al.* [23] used important landmarks such as the valves and ventricular septum cusps to act as control points to guide the automatic model fitting process for four-chamber heart modeling. Using a combination of prior shape knowledge in the form of an altas and manually placed landmarks, a generic model can often be deformed and used to map properties, such as Vadakkumpadan *et al.* [24] for fiber orientation and Vadakkumpadan *et al.* [25] for shape metrics, onto a reconstructed LV model using large deformation diffeomorphic metric mapping. However, both mapping approaches are restricted to the ventricular regions only.

One of the biggest obstacles in MR-based 4D full heart modeling has been the modeling of the passageways between the ventricle and the atrium and the complex vessels such as the pulmonary veins and aorta. This is largely due to the large inter-slice distance which creates strongly anisotropic voxels. During cardiac contraction, these vessels deform, possibly in directions perpendicular to the imaging planes. From the perspective of the short-axis image plane, this manifests as merging and splitting of the boundary of the region of interests, which causes difficulties during the 4D reconstruction process. To the best of our knowledge, no previous MR-based work has attempted to resolve this issue. In our work, we propose a methodology that aims to mitigate this issue by utilizing a tree structure to link and track the contours throughout the cardiac cycle, in order to reconstruct a 4D cardiac mesh model with 1-to-1 vertex correspondence.

## Overview of Method

**Figure 1:**
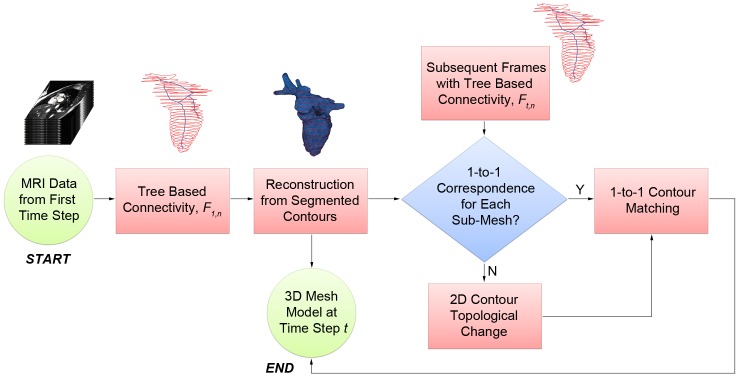
4D heart modeling algorithm flowchart. Using the segmented contours from the first time step, we go through each adjacent pairs of contours and establish the intra-connectivities between them. Using these connections, we reconstruct a 3D mesh model and set it as the generated mesh model of the first time step. For each subsequent time step, the segmented contours are passed through the tree-based connectivity process to establish their inter- and intra-connectivities. They are then compared with the mesh model from the current time step in a sub-mesh by sub-mesh manner. If there is no 1-to-1 contour correspondence, they are then passed through the 2D topological change process before performing the 1-to-1 contour matching process. Finally, the mesh model of the current time step is generated when the mesh is fully deformed.

The raw data input to this algorithm are the segmented contour lines, which are drawn on a set of short-axis MRI images over one cardiac cycle (see Figure 1). We establish a connectivity relationship between the images in two ways: (1) How the contours are connected to each other within the same time step, and (2) how the contours from one time step are connected to the contours in the next time step. Connectivities within the same time step relates to the morphological structure of the heart in that particular time step; connectivities across adjacent time steps provide information related to the motion of the heart across that time step.

To establish contour connectivities, both within and across time steps, a tree-based approach is proposed. Contours from the first time step are used to build an initial 3D model of the heart with a high mesh quality. The tree-based approach acts as a pre-filtering process to remove contour connectivities that are weak, which occurs due to the large spatial interval spacing between the MRI slices. For every subsequent time step, the set of segmented contour lines from that time step, together with its contour connectivities information, is matched with the current 3D mesh model to begin the deformation process, using a radial basis function approach. They are matched and deformed in a sub-mesh by sub-mesh manner, where a sub-mesh is part of the 3D mesh model, sandwiched by a pair of adjacent contours. Once all the sub-meshes are deformed in the 1-to-1 contour matching process, a 3D mesh model is then generated for that time step.

The main challenge occurs during *2D contour topological changes*, when there are no 1-to-1 connections between contours across time steps. In such an event, we have to determine both the direction of the vertical motion that the heart is undergoing, as well as the location of the 2D contour topological branching which manifests as a ridge feature in the 3D model. With both information, we apply a 2D sine-based deformation function using the ridge feature as its center to induce the vertical motion, and therefore ensuring that the contours can be associated in a 1-to-1 manner across that time step.

## Constructing a 3D model of the First Cardiac Frame

In this section we describe how we perform filtering to remove unwanted connections between segmented contours from adjacent slices, and then detail the reconstruction and refinement process of the 3D heart model, based on the MR images from the first time step. For clarity, we term each delineated border as a 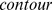, a set of non-intersecting contours lying on a plane as a 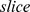, a set of slices that are parallel to each other and taken in the same time step as a 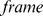, and a set of frames taken at different time steps as a 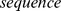.

### Model Definition

For convenience of explanation (but without loss of generality), we assume that each 2D contour is orientated such that it is lying in the 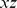-plane. As such, each slice is separated in the 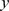-axis by an interval value 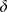, which corresponds to the inter-slice distance of the MRI data. The slices are indexed from 1, starting from the slice with the largest 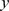-value, 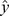. We define points 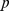 lying on the 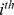 indexed slice as 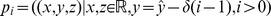 and contour 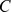 consisting of 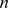 number of 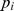 points such that
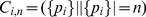
(1)A slice 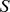 at index 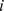 with 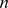 distinct contours is defined as
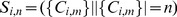
(2)A frame 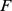 in time step 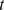 with 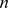 slices is defined as
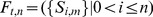
(3)Finally, a sequence 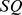 is with 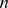 time steps is defined as
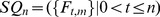
(4)


### Tree-Based Connectivity

There are two possible connectivity relationships that can be established between two distinct contours. *Intra-frame connectivities* can be formed between two contours from adjacent slices within the same time step, while *inter-frame connectivities* can be formed between two contours that are contained within the same slice index but from adjacent time steps. This entire set of connectivity relationships within a sequence is termed as a *tree-based connectivity*. The 3D surface reconstruction stage makes use of the intra-frame connectivities, while the deformation stage makes use of the inter-frame connectivities. In total, each contour can have up to four possible distinct types of connections, 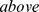 and 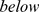, which are intra-frame connections; and 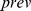 and 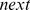, which are inter-frame connections. The four different connectivities type are defined by the following functions:
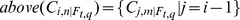


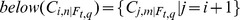
where 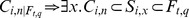, and
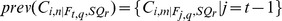


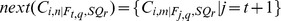
where 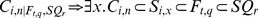. Note that the two sets of functions are the dual of each other, that is, if 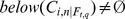, then 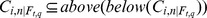. Similarly, if 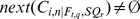, then 
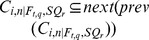
.

**Figure 2:**
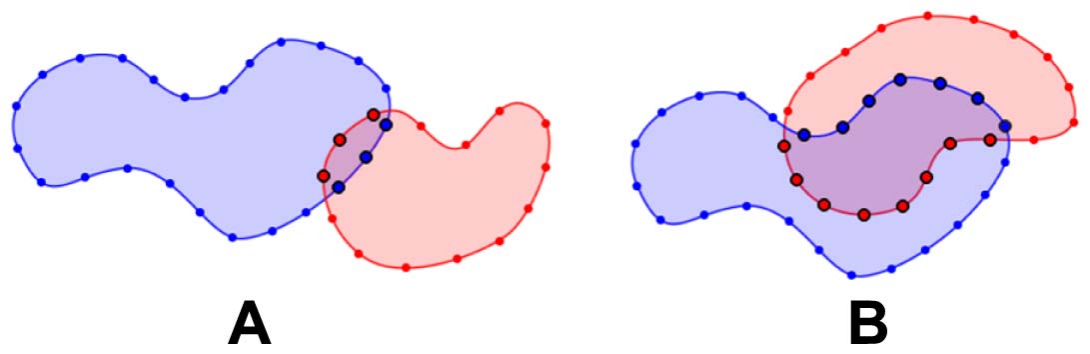
Determining connectivity between contours. In **A**, the red contour has a higher proportion (3 out of 14, 21.4%) of its perimeter lying inside the blue contour, as compared to the blue contour (3 out of 24, 12.5%) lying inside the red contour. However, the higher proportion is not enough to make a valid connection between the two contours. In **B**, the higher proportion of the two is 42.1% (8 out of 19), and a connection is thus formed between the two contours.

The tree-based connectivity is constructed by comparing each contour against all the other contours from the other adjacent slices and each pairing is given a similarity index. This similarity index measures the degree of overlap between the two contours when they are projected onto the same plane. We can compute this efficiently by finding the proportion that one contour lies within the interior of the other contour (in terms of perimeter), and vice versa. The larger of the two proportion values is then taken as the similarity index (see Figure 2). The similarity index can then be defined as
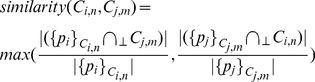
(5)where 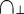 refers to a intersection operation based on a 2D orthogonal projection onto the 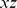-plane and 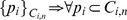. We can further expand the definition to include similarity index for slices, i.e.,
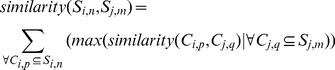
(6)


When all similarity indices between each distinct pairs of contours from both slices are obtained, the tree-based connectivity data structure is populated as follows: Intra-frame comparison fills in the functions for 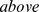 and 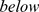, while inter-frame comparison fills in for the 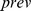 and 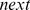 functions. In cases where there is only one pairing with non-zero similarity index, the assignment is straightforward. Otherwise, two situations could happen: Either 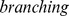 (intra-frame)/contour topological change (inter-frame) has occurred, or one of the similarity index is a very weak one. To decide between both potential situations, weak connections are filtered away first by setting a minimum similarity index threshold of 30% (see Figure 2). The underlying assumptions for selecting a threshold value of 30% is discussed in more detail in appendix S1.

Our pseudocode for establishing the intra-frame and inter-frame connectivities is as follows:

**Pseudocode:** *above & below*

**INPUT**: *C_i_*_,*n*__|F*_t,q_*_

**let** *S_above_* = *S_j,m_* **where** *S_j,m_*⊂*F_t,q_*, *j* = *i*−1

**for all** *C_above_*⊂*S_above_*

**compute** *current*←*similiarity*(*C_i_*_,*n*__|F*_t,q_*_,*C_above_*)

**if current** >0.3

*above*(*C_i_*_,*n*__|F*_t,q_*_)←*C_max_*

*below*(*C_max_*)←*C_i_*_,*n*__|F*_t,q_*_

**Pseudocode:** *prev & next*

**INPUT**: *C_i,n_*_|*F_t,q_*,*SQ_r_*_

**let** *S_prev_* = *S_i,m_* **where** *S_i,m_*⊂*F_j,q_*⊂*SQ_r_*, *j* = *t*−1

**for all** *C_prev_*⊂*S_prev_*

**compute** *current*←*similiarity*(*C_i,n_*_|*F_t,q_*,*SQ_r_*_,*C_prev_*)

**if** *current* >0.3

*prev*(*C_i,n_*_|*F_t,q_*,*SQ_r_*_)←*C_max_*

*next*(*C_max_*)←*C_i,n_*_|*F_t,q_*,*SQ_r_*_

**Figure 3:**
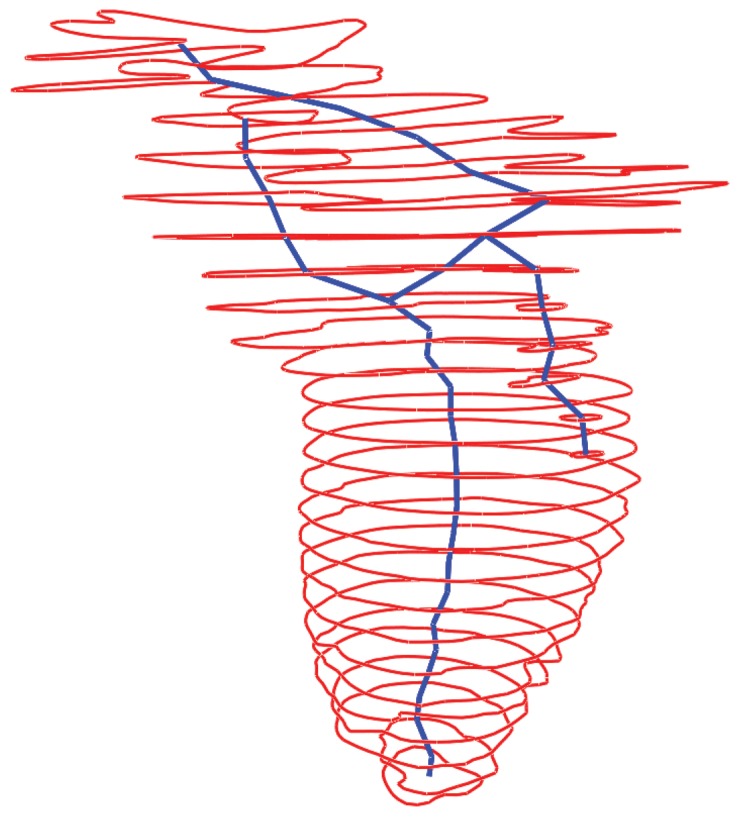
Tree-based connectivity data structure. The red lines depict the input contours from the MR images. The blue lines are drawn to represent intra-frame connectivities (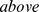 and 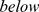) between the individual contour lines.

After weak connectivities are filtered off, we proceed to construct a final complete set of tree connectivity by using the concept of a minimum spanning tree. For intra-frame connectivites, the goal is to have an unbroken chain of connections that can traverse all the contours within a single frame. By representing the contours as nodes and the similarity indices as edges in a graph, we seek to construct a connected graph using the minimum number of connections. The only constraint is that those connections that are already established during the filtering stage are fixed and non-removable. A simple way to implement this is by using Kruskal's algorithm, where we sort all the edges from the highest similarity index value to the lowest. An edge that is not fixed is picked and if it connects two nodes that are unconnected, we add and form connections between the two nodes. Otherwise, the edge is removed. In this way, we can always establish a fully connected connectivity tree for the frame. A similar process is used for inter-frame connectivities, where we only consider contours from the same slice number throughout the entire sequence. Figure 3 illustrates the intra-frame connectivities for one single frame.

### Reconstruction from Segmented Contour Lines

**Figure 4:**
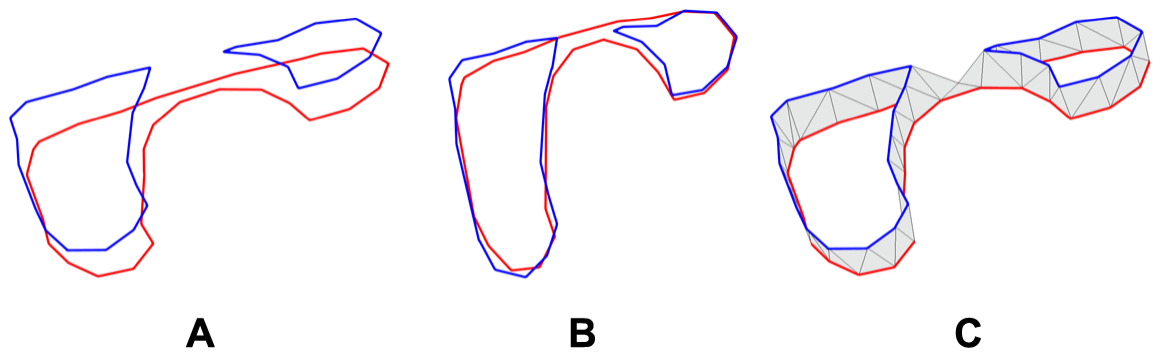
Surface reconstruction from segmented contours. In **A**, the two contours are shown in their original position in 3D perspective view. In **B**, the two contours are projected onto the same plane, and areas where they do not intersect are triangulated. In **C**, we can see the reconstructed surface between the two contours as they are restored to their initial position.

In this section, we describe our algorithm for surface reconstruction from segmented contour lines, which is improvised from the work of Barequet and Sharir [26]. The algorithm can be simplified to the problem of surface reconstruction between two sets of non-intersecting closed contour loops, where each set exists on a single plane and where both planes are parallel to each other (see Figure 4A). In order to create a surface that fits the contours from two adjacent slices, we first project the contours from both slices onto a common plane that is parallel to both slices (see Figure 4B). The internal area of the union of the these two contours consists of two different regions: Regions where the contours overlap, and regions where the contours do not overlap. We proceed to perform a boundary constrained delaunay triangulation of the regions where the contours do not overlap, by using points from the discretization of the contours. Since the two contours intersect, the interior of both contours are naturally split into different regions, and each non-overlapping region are bounded by perimeter segments from both contours. Thus, in each of the distinct non-overlapping region, the triangulation within will have triangles that consists of points from both contours. Note that for the top-most and bottom-most slices, we simply triangulate the interior of the slices in order to create a complete closed manifold. The last step of the reconstruction then is to separate the contours by projecting them back to their original planes. As some of the triangles contain vertices from both contours, they will naturally form a surface that wraps around both contours, hence resulting in the desired surface reconstruction between the contours (see Figure 4C).

**Figure 5:**
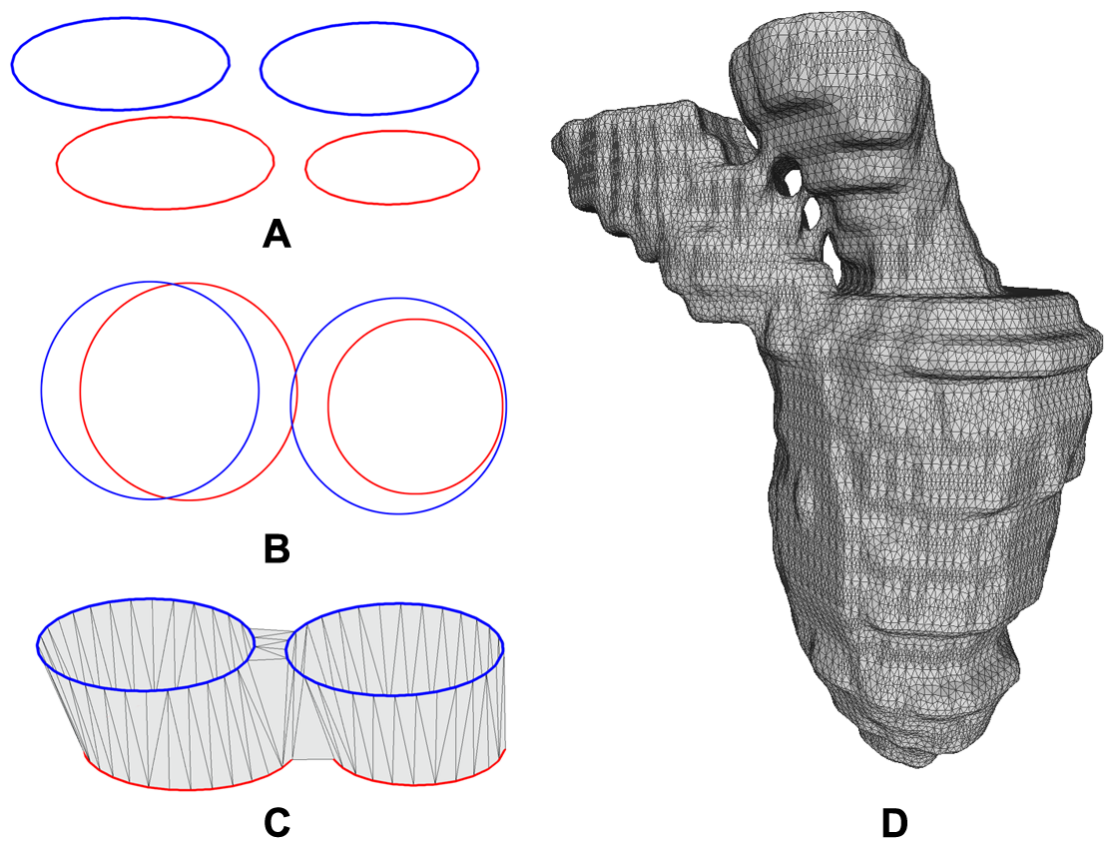
Common errors during reconstruction. In **A**, two sets of segmented contours are shown in 3D perspective, with the pair of blue contours from the same MRI image and the pair of red contours from another MRI image. In **B**, all four contours are projected onto the same plane. One of the blue contours is seen to have a very small intersection with a red contour. The resulting erroneous reconstruction is shown in **C**. In **D**, an example of such an erroneous surface reconstruction is shown when using the general marching cube algorithm.

There are two main issues arising from this approach. The first issue arises when there are minor intersections between the contours (see Figure 5). This can be resolved by using the tree-based connectivity data structure that was created *a priori* since we only need to form surfaces between contours that have connections defined in the connectivity data structure. The other issue is the possibility that some triangles in the reconstructed surface might have all three vertices belonging to the same contour, thereby resulting in a 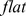 triangle after the contours are projected back. In the likely case that another flat triangle exists on the other side of the contour, the resulting surface will contain a collapsed triangle-pair, thereby forming a disconnected internal volume. In order to prevent such a problematic configuration, we inject new vertices into the triangulation before lifting. By using a chordal axis transform (CAT) approach [27], the new vertices introduced by the CAT essentially act as a set of “lifting” points to break up the flat triangles to prevent a collapsed configuration. These points not only help to create a relatively smoother volume but can also be used as interpolation points in the top-most and bottom-most slices to create a rounded tip.

### Surface Smoothing and Refinement

The large inter-slice interval 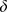 (with respect to the size of the human heart) of the MRI scan typically results in a reconstructed surface that is coarse, rough and potentially containing bad quality triangles. We improve the surface quality by an iterative two step process of first smoothing the surface to improve the average aspect ratio of surface triangles, and then increasing the vertex count by inserting additional vertices.

To achieve a volume preserving mesh smoothing with bounded error control, we adapt the well known 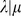 algorithm by Taubin *et al.* [28], [29], summarized by Equation (7). For each mesh vertex 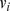, its new position 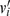 is determined by displacing the original position by the discrete laplacian multiplied by 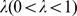 such that
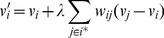
(7)where 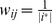, and 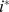 is the set of neighbors of 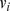. After updating all the vertex positions, a second pass is performed by replacing 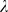 in Equation 7 with 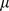 in which 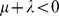 and 
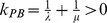
, where 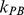 is the pass-band frequency. Typically, 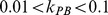 produces good results.

Applying smoothing will inevitably result in vertices shifting away from the original surface mesh model. To control the error introduced, we devise an 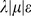 algorithm that incorporates an error correction scheme by taking into account the deviation of each vertex from its normal plane before smoothing. The aim is to prevent the smoothing algorithm from modifying the position of the vertex beyond a certain tolerance 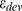 from its normal plane. The unit normal vector 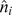 at vertex 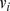 is defined using the scheme proposed by Max [30]. Next, we find the deviation vector 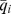 such that 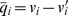. Then, 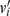 is conditionally modified such that,
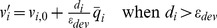
where the deviation from the normal plane, 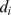, is equal to 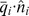. Note that this error correction is performed for both the 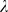 and 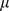 passes of the smoothing scheme.

To upsample the model, new vertices must be added to the mesh model. However, without an original base model, the position of new vertices can be hard to determine. To resolve this issue, we use the approach by Su and Kumar [31] for refining the mesh model. For each triangle 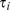 with vertex normals {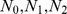}, we fit a quartic Bézier patch 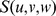 over it such that
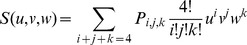
(8)where 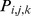 are the control points of 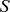 and 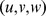 are the Barycentric coordinates associated with 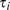. Essentially, for a point 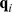 lying on 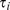, we compute the barycentric coordinates of 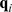 in 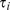 and then use these barycentric coordinates to compute the compensated position of 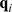 in the space of 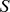. In doing so, the geometry could be faithfully preserved throughout the refinement process.

## 4D Morphing of the Heart Model

The initial 3D model of the heart is reconstructed based on the contours taken from the first frame. Using the contours from subsequent frames and their corresponding tree-based connectivity, we deform the initial heart model to conform to the contours in the subsequent frames. This process is performed iteratively until we achieved a set of 3D models that has 1-to-1 vertex correspondence, and this forms the 4D heart model. In this section, we explain the methodology to perform such a deformation.

### Methodology of the Deformation Process

**Figure 6:**
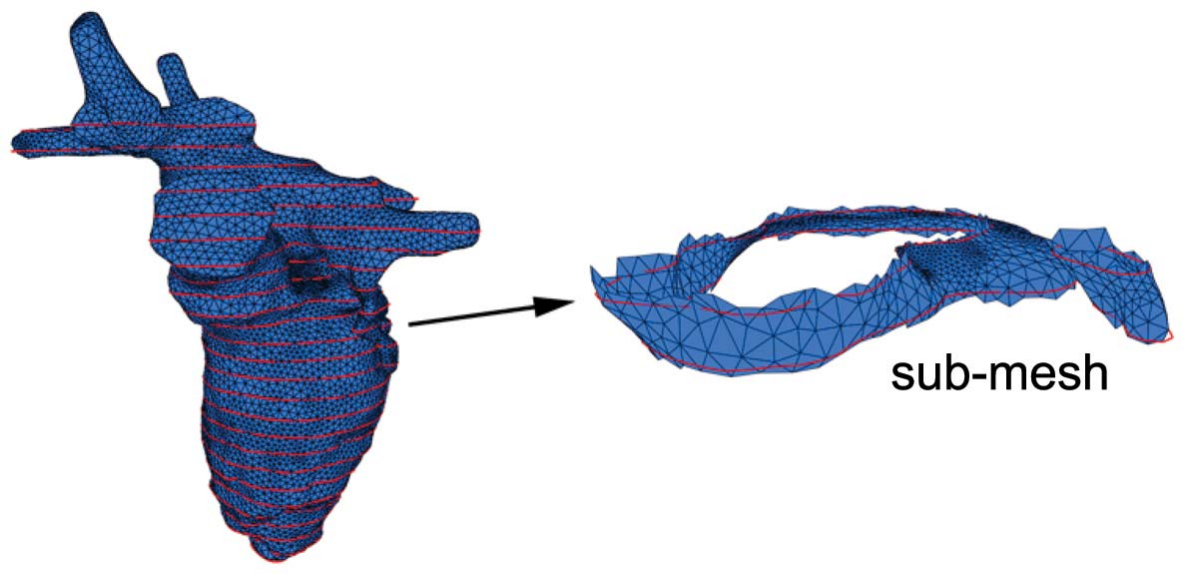
Sub-mesh. The image on the left shows the 3D mesh model of the left heart that is deformed to fit the contour lines (shown in red). The image on the right shows a sub-mesh that is bounded by a pair of contour lines.

The goal of the deformation process is to modify the heart model of a particular frame to fit the contours of the next frame without changing the mesh connectivity. To implement the deformation process, we split the heart model into logical sub-meshes using the MRI planes as the partitioning planes. As each sub-mesh (except the topmost and bottommost) is sandwiched between two partitioning planes (see Figure 6), we can compute the deformation for each sub-mesh independently. Using an example for illustration, consider two adjacent slices 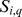 and 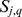, where 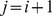 and 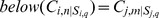, where each contains just a single contour (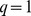), and a surface 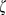 between them. Considering just 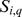, we use 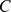 to represent 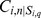 and 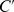 to represent 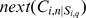.

Taking these two contours 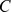 and 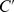, we wish to find a mapping of the points on 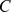 (source points) onto the contour 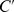 (target points). Note that the target point set does not have to correspond to the actual points on 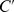, rather they just need to lie on the 2D polygon, as defined by the points on 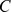. The detailed process to achieve this mapping will be described in the next section. Using both sets of source points and target points, we generate a radial basis function (RBF) interpolant [32] of the form
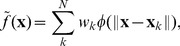
(9)where the kernel function 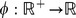 is univariate and radially symmetric, and 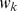 is a set of 3-dimensional weights. Our choice of the kernel function is the 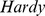 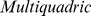, 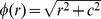, where 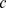 is a constant defined individually for each source point, based on the distance to its closest neighbor. By determining the weights of the RBF, we are able use it to interpolate where the new surface 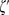 lies on.

The advantage of using RBF is that there is a 1-to-1 correspondence between the vertices from 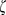 to 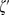. For the case where there is only one sandwiching slice for the sub-meshes, we find a vertex that is the furthest away from the plane of the slice, and add that point to both the set of source point and target point. This prevents the source-target set to have only points residing in two dimensions, which will otherwise result in singularity issues for the RBF computation. In this standard deformation process, we assume that the number of contours in 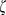 is equal to 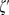 for both the top and bottom slices of 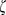. However, it is possible to have 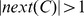 or 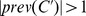, which is a situation that we termed as *2D contour topological change* and has to be handled separately.

### Contour Matching

Using 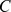 and 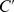, we wish to map the points in 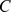 onto the polygon as defined by the points in 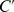. In doing so, we want to minimize the distance of the mapping for the points and to avoid any crisscrossing of the motion paths (source to target paths) of the points. Minimizing the mapping distance prevents distortion from occurring in the new surface 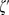. Moreover, avoiding crisscrossing of motion paths will prevent singularity issues during RBF transformation.

**Figure 7:**
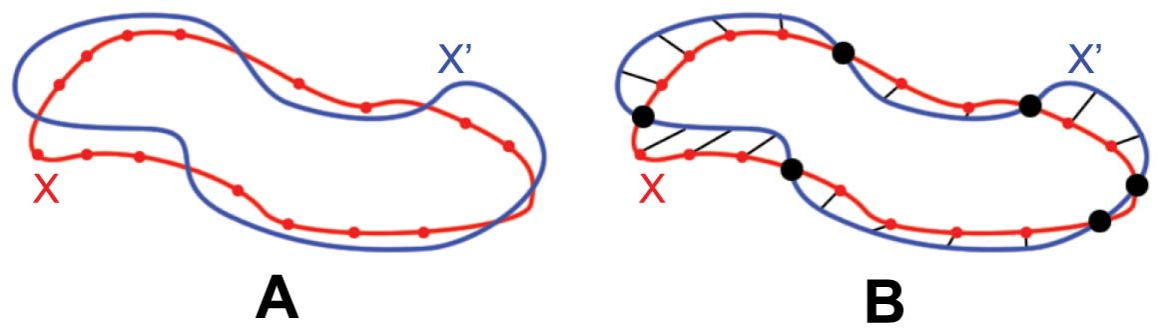
Contour matching for generating a source-target point set. In **A**, we wish to map contour 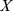 (shown in red) to contour 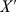. Both contours are mapped and projected onto the same plane. In **B**, the intersection points between both contours are found and is used to split both contours into segments. The segments are then compared to generate the source-target point set.

To achieve a good mapping, we project 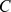 and 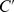 onto a common plane. Next, the intersection points between 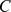 and 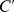 are then used as breaking points to split both 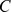 and 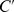 into segments. Each individual segment in 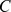 is then matched against its corresponding segment in 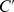, and the points in each segment are then mapped from 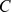 to 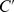 proportionally. From empirical findings, four breaking points are usually more than enough to adequately perform the matching process (see Figure 7). To avoid crisscrossing of motion paths, we further divide up each time step 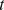 into 10 equal time steps 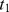 to 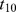 and similarly with the motion path of each point. With the smaller time step, the motion paths of the source set are less likely to cross each other.

### Handling 2D Contour Topological Changes

A 2D contour topological change occurs when there is no 1-to-1 matching of contours during the contour matching process, i.e., 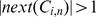 or 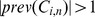. For such situations, we first determine the direction of the vertical motion that is occurring at the contour, then locate the ridge feature, and finally apply a 2D sine-based deformation function centralized at the ridge feature. The goal is to make sure that there is a 1-to-1 matching of all the contours between the current frame and the next frame.

#### Determining Vertical Motion

Vertical motion is deemed to have occurred when the number of contours on the same slice differs from the next time step, resulting in a 2D topological change of the contours on that slice. This slice is referred to as the 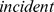 slice (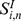), where a straightforward contour matching and deformation process will not work, since a one-to-one correspondence between the contours does not exist.

To resolve this, we need to ascertain the direction of the vertical motion using a heuristic as follows: To determine the type of vertical motion for the case of 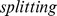 change, where 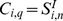 and 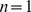:
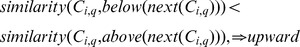


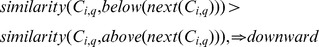
To determine the type of vertical motion for the case of 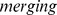 change, where 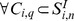 and 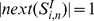:
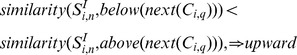


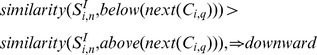
For the case of upward vertical motion, the slice below the incident slice is referred to as the 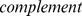 slice, i.e., 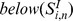. For the case of a downward vertical motion, the complement slice is the slice above the incident slice, i.e., 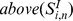.

#### Ridge Detection

Before we can apply a vertical deformation around the vicinity of the 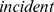 slice, we need to determine where the branching of contours has occurred, which is at a *ridge feature*. A ridge feature is a path described by a sequence of edges that are roughly equidistant from the two contours. The desired effect is to apply a stronger vertical deformation at the source of the branching, while gradually reducing the strength as we move further away from it. We use a 2D sine-based function, with its center localized along the ridge feature, as the vertical deformation function.

**Figure 8:**
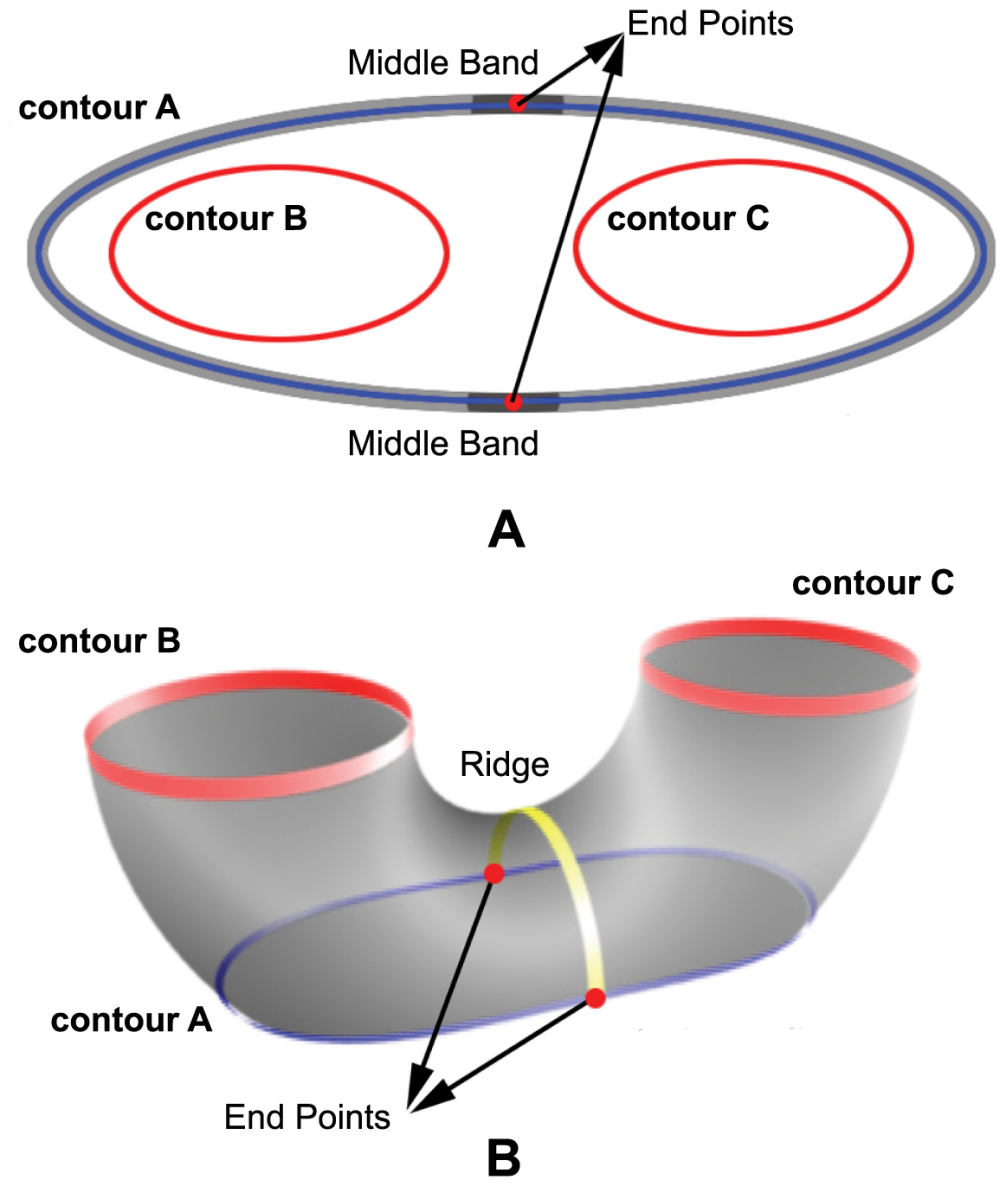
Ridge detection. In **A**, contour 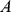 is broken down into three different bands. Within the middle band, two points are chosen to mark the start and end points of the ridge. In **B**, a ridge is represented by the yellow path that traverse between the start and end points from the middle band of contour 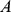.

To extract the ridge, we have to first locate the two end points of the ridge. Assuming that the incident slice contains contour 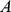 and the complement slice contains two contours, 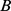 and 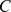, we proceed to project all the three contours onto a common plane. For each vertex point on 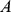, we compute its nearest distance to both 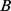 and 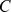. For vertex points where its nearest distance to both 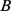 and 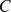 do not differ by more than 10%, we term it as the middle band (see Figure 8A). Typically, two contiguous and disjointed middle bands will be formed. However, in the unlikely case where multiple contiguous middle bands are formed, we select the two largest bands and discard the rest. A point in each band is identified such that the difference between its distance to both 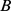 and 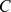 is the smallest. These two points are then selected as the end points of the ridge. The ridge is represented by the geodesic path computed between these two end points. A simple greedy algorithm that always selects the next edge such that it is closer to the end location is sufficient to extract the ridge. Figure 8B shows an idealized image of the ridge feature that is formed between two adjacent slices.

#### Applying Vertical Motion

To induce smooth vertical deformation, we have to expand the affected regions to include other sub-meshes around the incident slice. This allows the deformation to gradually spread its effect across several slices, thereby creating a smoother transition to the next frame. Hence, we considered two layers of adjacent sub-meshes above and below the incident sub-mesh. In total, six different slices are affected, labeled from 1 (topmost) to 6 (bottom). The deformation employs a 2D sine function, with its center located along the ridge feature. This allows us to apply the largest deformation at the ridge feature, while gradually reducing its effects, as we move away from the ridge.

We proceed to project all the affected contours located on the six slices onto a common perpendicular plane, together with the ridge feature. On this perpendicular projection, the parameter 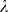 is set to be the furthest 2D distance between the ridge and any point on the incident slice. We then use a 2D sine function to compute the amount of vertical displacement 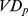 of each point 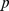 on the contour as a function of its 2D distance to the ridge 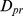.
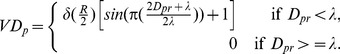
(10)The value 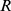 acts as a control variable to adjust the amount of deformation experienced by each individual contour. An 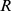 value of 1 shifts a vertex by 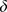 upwards, while an 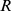 value of −1 does the opposite. By setting 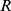 independently, as illustrated in Table 1, we can then adjust and smooth the effect of the deformation across the slices. After inducing the vertical motion, a 1-to-1 correspondence can be established for each slice between the current time step and the next. We can then proceed using the standard deformation process to deform the mesh into the next time step.

**Table 1:** Listing of applied R values.

	*R* value	
Slice Position	Upward Motion	Downward Motion
1	0.2	−0.2
2	0.4	−0.5
3	0.7 (Incident)	−1.2 (Complement)
4	1.2 (Complement)	−0.7 (Incident)
5	0.5	−0.4
6	0.2	−0.2

The amount of 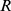 value applied to the different slices adjacent to both the incident and complement slices for both upward and downward motion cases.

## Results and Discussion

We implemented the 4D heart deformation algorithm in C++ (with no multi-threading optimization) on an i7 core 3.07 GHz machine and tested it on five sets of MRI scanned data of the left heart of five healthy patients. An additional dataset consisting of an idealized model of the left heart is included as a reference comparison. The MRI data was acquired using steady state free precession sequence with retrospective electrocardiographic gating, and consisted of contiguous images covering the left atrium to the apex of the left ventricle. As the datasets are obtained from different sources, they differ in terms of the slice thickness of images and time difference between frames. Hence, the number of slices required to cover the left heart and the number of frames required for one heartbeat is different for each dataset. The contours of the endocardial surface are segmented manually by a cardiologist using the CMRTools software developed by Cardiovascular Imaging Solutions Ltd. These segmented contours are then used as input for our testing purposes.

### Experimental Setup

**Figure 9:**
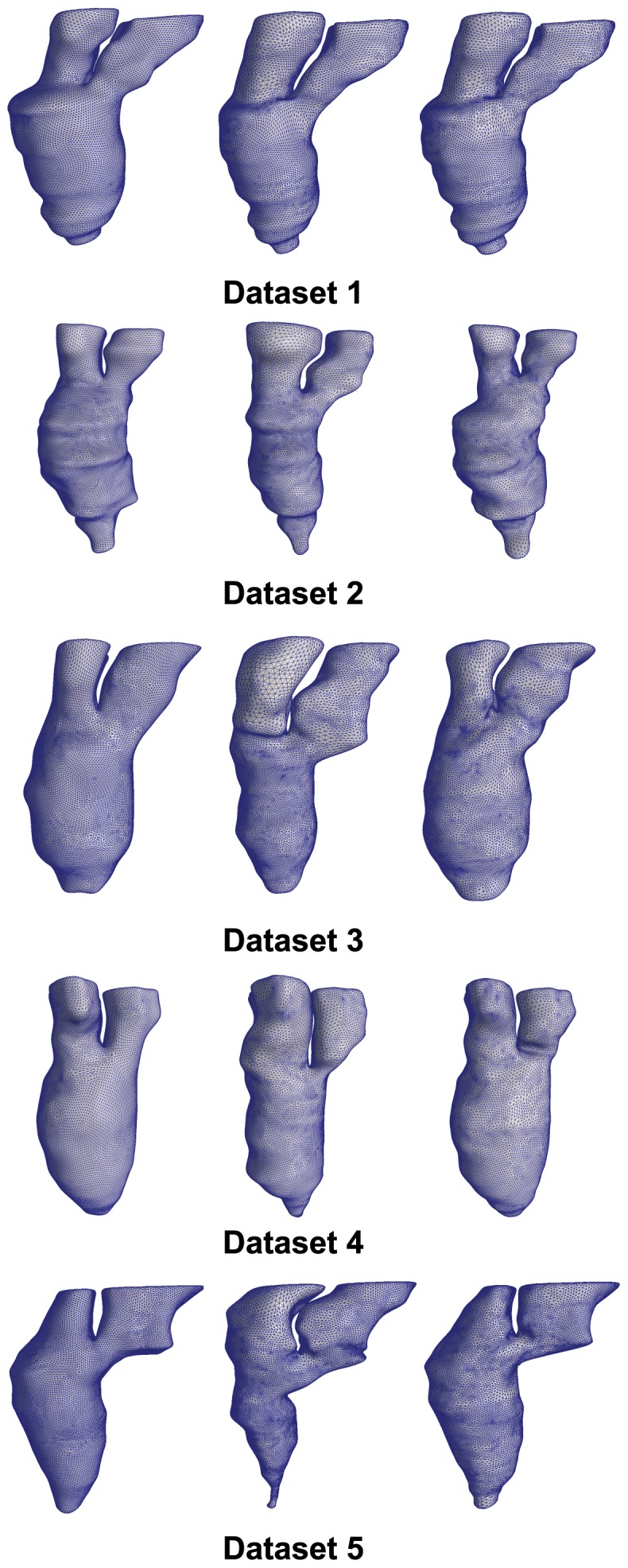
Reconstructed 4D heart model. For each dataset, we select and show three of its 3D surface models. The left model is taken from the first frame, the center model is selected from the middle frame, while the right model is taken from the last frame.

For each patient dataset, we apply the 3D reconstruction algorithm on the input contours taken from the first frame to generate a conforming 3D mesh model of the left heart. Our input contours delineate the full left ventricle, the left atrium and part of the aorta. Using surface smoothing and refinement techniques described previously, the 3D mesh models are refined to achieve a realistic 3D surface mesh of the left heart. A table listing the profile of each dataset and its reconstructed 3D mesh model is shown in Table 2. Using this refined 3D mesh model, a set of 3D mesh models is generated to form a 4D heart model of the left heart, based on the deformation algorithm (see Figure 9). Each individual 3D mesh model in the generated set conforms to the stack of segmented contours lines at its corresponding frame. Additionally, the set of 3D mesh models maintains vertex correspondence throughout.

**Table 2:** Dataset Profile.

Dataset No.	Slice Resolution (mm)	Vertex Count	Triangle Count	No. of Frames	No. of Slices	No. of Frames with Topological Changes
1	1.77×1.77×5	17260	34516	24	24	4
2	1.77×1.77×10	19456	38908	24	15	2
3	1.77×1.77×10	20429	40854	24	12	2
4	1.45×1.45×8	17757	35510	21	12	2
5	1.45×1.45×8	21384	42764	21	14	2

Profile of the 5 datasets used for testing.

Our goal is to generate a set of good quality mesh models that conforms to the given segmented contours over all the frames covering one cardiac cycle. Our experimental setup therefore consists of measuring the conformity of the mesh model to the given segmented contours and the quality of the mesh as it deforms from frame to frame. Lastly, we provide the computational time of each dataset as an indicator of the performance of the algorithm.

### Mesh Conformity

**Figure 10:**
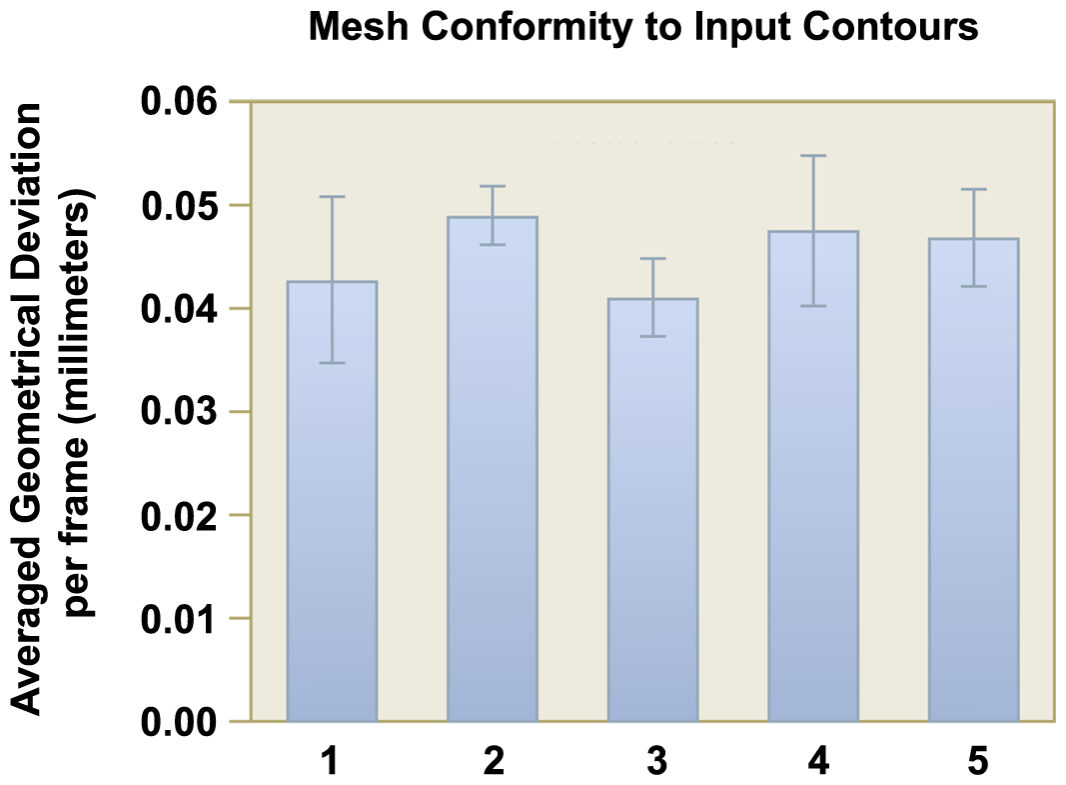
Mesh conformity with input segmented contours. Each frame of the generated 3D mesh model for each dataset is tested for its fidelity to the input contours. The mean distance separation over all frames for each dataset is shown in this chart. The standard deviation of the separation is shown as a line bar for each dataset.

For each dataset, we measure the conformity of the set of 3D mesh models against the input segmented contours at each corresponding frame in terms of absolute geometrical deviation. The comparison is done by taking measurements at a constant interval along the input segmented contours. Based on our experience, a constant interval of 0.2 mm is sufficient for our purpose as a higher sampling will not affect the final value of average distance separation between contour and mesh. At each interval point on the input segmented contour, we measure its distance to the nearest point on the 3D mesh model. We then take the average of all the geometrical deviations of all the sampling points in each frame. Finally for each dataset, we tabulate the mean and standard deviation of the averaged geometrical deviation of all the frames. The results for the five datasets are shown in Figure 10.

As observed in the figure, although the slice difference for the datasets ranges from 5 mm to 10 mm, the average geometrical deviation between the contours and the mesh models across datasets are similar, which is around 0.041 mm to 0.049 mm (maximum is around 0.047 mm to 0.057 mm). Taken as a percentage of the slice difference, the average separation lies between 0.41% to 0.85% of the slice difference. This is a very small value and it shows that the geometry of the 3D mesh models conforms very well to the input segmented contours.

**Figure 11:**
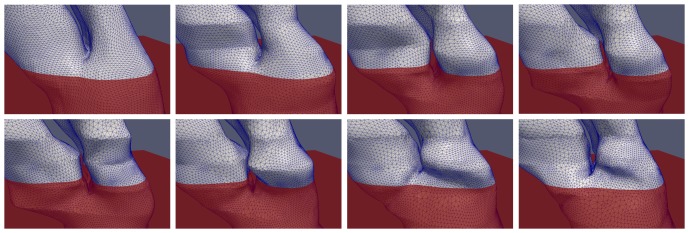
Illustration of inter-frame 2D contour topology changes in reconstructed 4D heart model. A sequence of images (left to right, top to bottom) depicting the modeling of 2D contour topological deformation. A red plane is added as a reference, to illustrate the change in the number of contours over that plane throughout the cardiac cycle. The contours (the intersection between the red reference plane and the heart model) are observed to merge and split again.

For the case of 2D contour topological changes, we illustrate the conformity of our 3D mesh models to the slices as it undergoes topological change in a series of screen shots taken from Dataset 1 (see Figure 11). A red plane depicting the scanning plane of the MRI is shown to intersect with the generated 3D mesh model. In addition, we are able to observe the distinct merging and splitting of contour(s) on the red plane, which is consistent with the drawn contours in the dataset.

### Mesh Quality

Maintaining vertex correspondence is one of the key objectives of the 4D heart deformation algorithm as the 3D mesh model deforms from frame to frame. Moreover, the algorithm has to constantly ensure that the mesh quality is of an acceptable quality at each time step. Generally, the angles of the triangles within the mesh give an indication of the mesh quality. There are two indicators that we measure in this experimental setup. One is the percentage of triangle angles that lie within the range of 40 to 80 degrees, which is typically considered to be within good quality range. In addition, we track this percentage value as the mesh deforms from the first frame to the mid frame, and finally to the last frame. The other indicator that we measure is the number of bad quality triangles which occur per frame. A bad quality triangle is one which contains an angle of less than 25 degrees.

In Table 3, the percentages of good quality triangles in the five datasets are shown. This includes a breakdown of values into smaller 10 degree ranges from 40 to 80 degrees. We observe from the table that the distribution of angles remained fairly consistent as the mesh deforms over all the frames. This is in spite of the fact that large deformation occurs at frames with slices undergoing 2D contour topological changes. Overall, 80% to 90% of the angles are within the range of good quality.

**Table 3:** Distribution of Triangle Angle Quality.

Dataset No.		% of angles (in degrees) between				
		40–50	50–60	60–70	70–80	Total
1	First Frame	18.54%	26.68%	21.53%	15.68%	82.45%
	Mid Frame	18.48%	26.09%	24.47%	16.56%	85.62%
	Last Frame	18.02%	27.09%	26.24%	16.04%	87.41%
2	First Frame	16.34%	30.77%	31.84%	13.59%	92.56%
	Mid Frame	16.61%	31.71%	27.73%	14.97%	91.04%
	Last Frame	17.73%	29.95%	28.77%	14.73%	91.20%
3	First Frame	15.33%	31.25%	32.87%	13.51%	92.98%
	Mid Frame	17.25%	30.78%	28.53%	15.05%	91.62%
	Last Frame	16.27%	32.77%	28.58%	14.65%	92.29%
4	First Frame	16.07%	31.86%	31.38%	13.94%	93.26%
	Mid Frame	17.70%	30.25%	27.44%	16.05%	91.46%
	Last Frame	16.33%	31.51%	29.57%	14.42%	91.85%
5	First Frame	14.95%	31.46%	34.05%	13.20%	93.68%
	Mid Frame	19.25%	26.27%	26.47%	17.20%	89.21%
	Last Frame	18.77%	28.61%	25.88%	16.21%	89.49%

This table shows the percentage distribution of triangles with angles that are within good and acceptable ranges. In addition, the table listed the distribution patterns within each dataset as it transits from the first frame to the middle frame and finally to the last frame.

In addition, the rate of occurrence of bad quality triangles is shown in Table 4. From the results, we noted that their rate of occurrence is in fact very low: Most of the datasets have an average of less than one bad quality triangle per frame. Dataset 1, however, has a higher occurrence rate of 2.708 per frame. This is because it has more slices (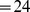) per frame due to the fact that it has the smallest slice thickness among the datasets. These slices restrict the motion of the 3D mesh models as it is actually a constraint on the shape of the 3D mesh model. As there is less leeway for the triangles to be smoothed out, the occurrence rate of bad quality triangle generally tends to increase. Nevertheless, the percentage occurrences of bad quality triangles is still very low (ranging from 0.0009% to 0.007%) for the five tested datasets.

**Table 4:** Occurrence of bad quality triangles.

	Dataset No.				
	1	2	3	4	5
No. of bad quality triangles per frame	2.708	0.875	0.375	0.333	0.875
% occurance	0.0078%	0.0022%	0.00091%	0.00093%	0.0020%

This table shows the average and percentage occurrence of triangle with low aspect ratio per frame for each dataset. A triangle containing an angle lower than 25 degree is considered to be of bad quality.

### Computational Time

**Figure 12:**
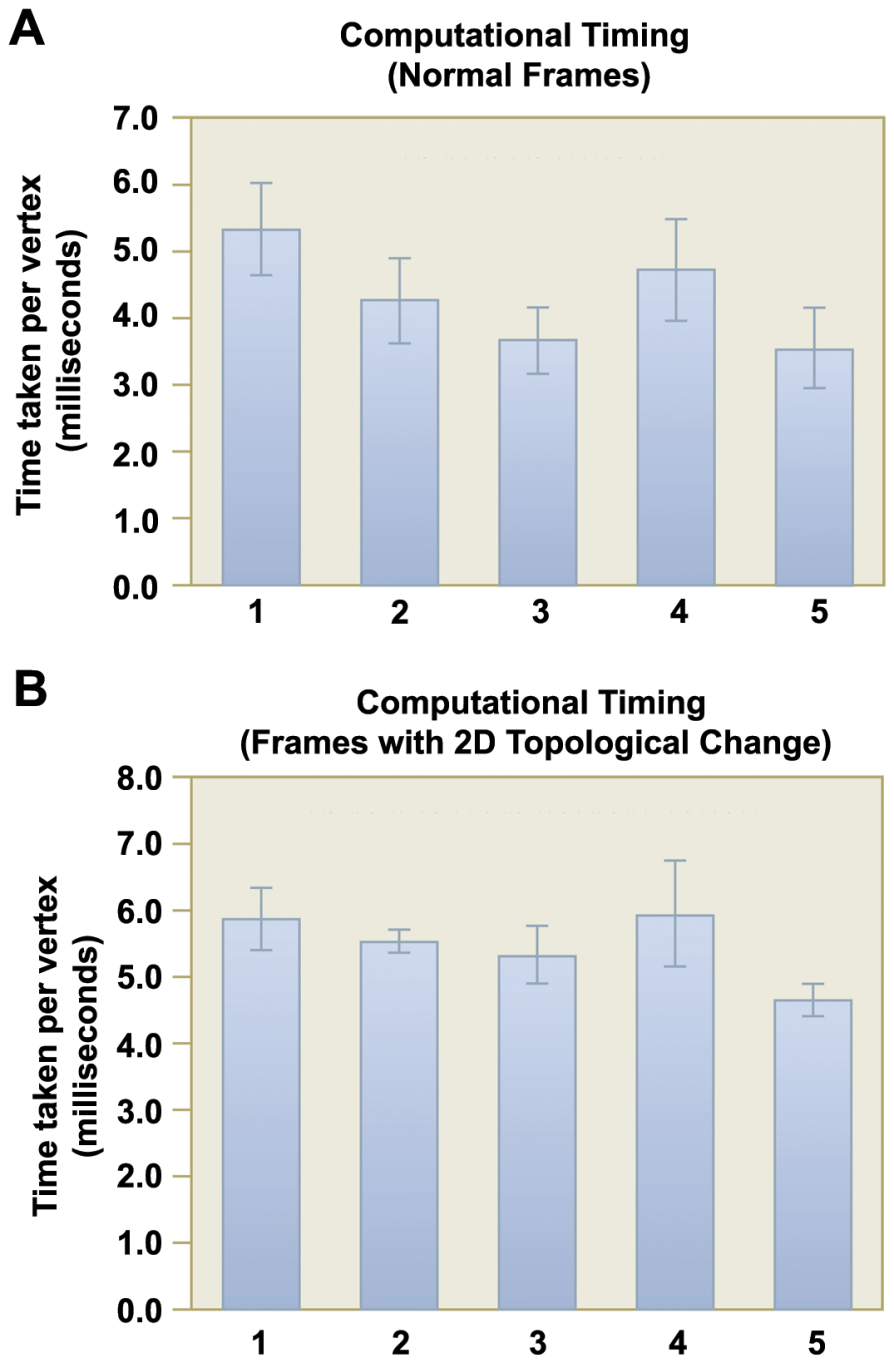
Computational time performance . In **A**, the mean CPU time (in milliseconds) incurred per vertex for normal frames in each dataset is shown. In **B**, the same information for frames with 2D contour topological changes is shown. The standard deviation in CPU time across different frames is shown as a line bar for each dataset.

We present the results of the computational performance in two graphs: Figure 12A shows the average CPU time taken per vertex for normal frames, while Figure 12B shows the average CPU time taken per vertex for frames that have topological changes occurring in them. The standard deviation for each dataset is also shown as a black bar on the data chart. Generally, the average computational time is around 4.5 milliseconds per vertex for normal frames, and 6 milliseconds per vertex for frames with 2D contour topological changes. There is little timing variability among frames, both within and across datasets. Typically, for a 3D mesh model with a vertex count of 20000, it would take approximately 1.5 minutes to complete a frame. To generate a 4D heart model consisting of 20 frames would take around half an hour to complete, which is considered to be adequate for practical usage. We acknowledge that further optimization can be made to further improve its performance, such as inclusion of parallel programming which can exploit the natural compartmentalization of computational load for each sub-mesh.

### Comparison with Idealized Model

**Figure 13:**
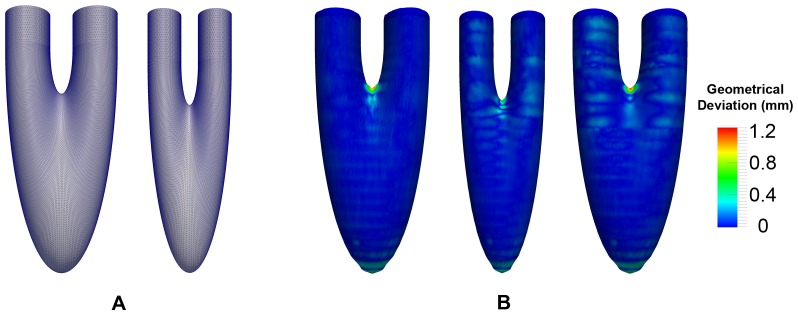
Idealized Dataset. In **A**, the idealized mesh models for the end-diastolic state (47040 vertices) and the end-systolic state (34400 vertices) of the left heart are shown. Using these models, we extracted segmented contours from them at a regular slice interval of 5 mm. The segmented contours are then used as input to our algorithm over three frames (first and last frame are set as the end-diastolic state, while the middle frame is set as the end-systolic state). The generated mesh models for the three frames, colored based on the geometrical deviation per vertex, are shown in **B**.

For all the patient datasets, the inputs are a set of segmented contours obtained from the MRI images. As such, when measuring the conformity of the 3D generated models, we are only able to compare them with the set of segmented contours. As a point of reference, we include an additional dataset where the input is extracted from an idealized 4D mesh model of the left heart. This dataset consists of three frames. The first and last frame is exactly the same, and they depict the state of the left heart in the end-diastolic state. The middle state depicts the end-systolic state of the left heart. The surface of the idealized model is constructed by first sweeping an ellipse (major axis of 2 units in the 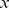-axis and minor axis of 1 unit in the 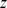-axis) centered at (3,0,0), 180 degrees clockwise about the 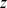-axis. The whole model is then translated such that the saddle point is exactly at the origin. The end-diastolic (first and last frame) state is formed by scaling the idealized model using a scaling factor of (5,20,18), while the end-systolic (middle frame) state is similarly formed by using a scaling factor of (3.5, 18.75, 14). The end-systolic state is then further translated by (0,−5,0) to account for the vertical shortening with respect to the end-diastolic state, which results in 2D topological changes between each frame. For both states, the boundaries of the model (2 ellipses) is then extruded upwards in the 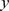-axis to a height of 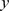 = 35. Finally, the models are finely meshed with a vertex count of 47040 and 34400, respectively, and presented in Figure 13A. Using a planar intersection with the mesh models at an interval of 5 units, we are able to extract a set of segmented contours that is similar to the set obtained from the five patient datasets and run it through our algorithm. The 4D model generated using our method is then compared with the idealized model for geometrical conformity, shown in Figure 13B. The tabulated results are listed in Table 5.

**Table 5:** Comparison with idealized model dataset.

Frame No.	3D Model			Segmented Contours		
	Mean	Max	RMS	Mean	Max	RMS
1	0.074	1.195	0.107	0.030	0.443	0.032
2	0.078	0.917	0.103	0.045	0.392	0.045
3	0.081	1.244	0.115	0.036	0.316	0.033

This table shows the Hausdorff distance of our generated model with the idealized mesh model. The results of comparing with the segmented contours are also included for reference. All values are in millimeters (mm).

The results showed that in the three frames, with two of them having 2D contour topological changes, the mean Hausdorff distance ranges from 0.074 mm to 0.081 mm, which is around 1.48% to 1.62% of the inter-slice distance. These values, although higher than the mean difference compared with the segmented contours, are still relatively low and indicate a good approximation to the original idealized model. It was observed that regions of higher errors tends to occur at the saddle and apical region. For the apical region, the profile is approximated by a series of points that are generated using the CAT approach described in the earlier section. These points are pulled downwards to generate the shape of the apical cap, which resulted in a flatter tip as compared to the original ellipsoid. For the saddle region, its profile is determined by the ridge, which was extracted and deformed during the 2D topological change deformation stage. After deformation, the profile of the ridge tends to deviates significantly from the original smooth profile of the ellipsoid surface.

### Limitations and Future Work

Indeed, we recognize that the current limitation of our approach is the lack of incorporation of the twisting motion of the heart. To our best understanding, the gold standard in extracting myocardium twisting is via tagged MRI [11], [33], albeit that the method still suffers from tag fading. Nevertheless, a lack of this twisting motion does not necessary mean that it is irrelevant to computational physiological modeling. As shown in [16], a hexahedral grid representing myocardium motion was generated and employed for time dependent simulations of blood flow in human left ventricle. Even though twisting motion was not incorporated in the model, the numerical results showed some realistic prediction of the intra-ventricular flow pattern in comparison with data obtained from MRI flux measurement. However, it is essential for boundary-prescribed computational fluid dynamics simulations to have a 1-to-1 vertex correspondence or a consistent surface mesh topology in order to perform the hemodynamic computation, which is what our method can offer. In fact, it is usually the mesh generation portion of the simulation workflow that creates the bottleneck, and our method could serve to shorten the overall simulation turnaround time. Hence, we believe that our proposed approach has utility in actual computational applications. In addition, our approach can potentially be refined in the future by incorporating more accurate motion-related information in the RBF routine of the algorithm, especially when motion imaging modality (such as tagged MRI) improves.

Another point of discussion is concerning the input to our 4D heart deformation algorithm, which is the set of manually segmented contour lines that are drawn across all slices throughout the cardiac cycle. This is a laborious task which might take hours to complete even for a skilled practitioner. Our future goal is to incorporate a robust image segmentation algorithm into our work. There is a huge pool of literature on automatic image segmentation for CMR data. While these “automatic” solutions might not be foolproof under actual clinical conditions, they can already serve to speed-up the segmentation task significantly. In clinical research using cardiac MRI data, the segmentation task can be performed using a semi-automatic approach with slight user intervention to great effect. This is feasible because MRI has much fewer slices as compared to CT. In principle, it does not matter where the segmented contours are obtained from, as it is not the focus of this work.

**Figure 14:**
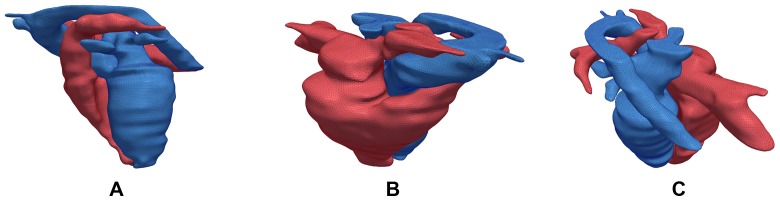
Full Heart Model. Using Dataset 1, we created a full 4D heart model which consists of both the left and right heart. The left heart is shown in blue and the right heart is shown in red. In **A**, we present the view with the left heart in front. In **B**, we present an angled view from the top depicting the right heart in front. In **C**, another top angled view from a different perspective is presented. All three views are taken from the first frame.

Our 4D models are created based on geometrical deformation, in a sub-mesh by sub-mesh, frame by frame manner. This allows us to maintain a high level of conformity to the input contours (average of 0.04 mm to 0.05 mm). However, it has also resulted in the mesh having a slight “stair-case” look. Part of the reason for this could be due to patient movement during image capturing. We can further mitigate some of it by using more advanced mesh smoothing techniques. A possible approach might be using Hermite functions [34] to create smooth interpolated representation of the ventricles, using an image registration based method developed by Barber *et al.* [35] to deform the template mesh to other patient MRI data. However, to achieve that level of smoothness, a compromise will have to be made in terms of conformity to the input data (average of 0.37 mm to 1.42 mm). This technique is further extended by Zhang *et al.* [36], who use CT data along with some user interaction to trace out a center-line path (similar to our tree-based connectivity), to create a full heart model model that can be deformed to fit other patient CT data. As a point of comparison, we created a detailed and smoothed model of the 4D full heart model based on Dataset 1, consisting of both the left and right heart. Several views of the first frame of the 4D full heart model is presented in Figure 14.

This methodology can be easily extended to other human anatomies with dynamic motion, such as the lungs, or be utilized as a base mesh model that can be used to quickly generate other patient's heart model through the deformation process. In essence, as long as the cross-sectional area of anatomy's internal region captured on the 2D MRI image is not relatively small as compared to the MRI inter-slice distance, for example those of the blood vessels, this methodology should still be applicable.

## Conclusion

In this paper, we presented a methodology for the automatic 4D reconstruction of a patient specific cardiac mesh model using segmented contour lines from MRI images. The methodology is able to handle inter-frame and intra-frame 2D contour topological changes which occur during the course of the cardiac cycle. The output is a sequence of high quality 3D mesh models with 1-to-1 vertex correspondence that depict the cardiac shape at each time step. Such a model is useful for downstream computational simulation purposes.

## Appendix

### Determining Similarity Index Threshold

**Figure 15:**
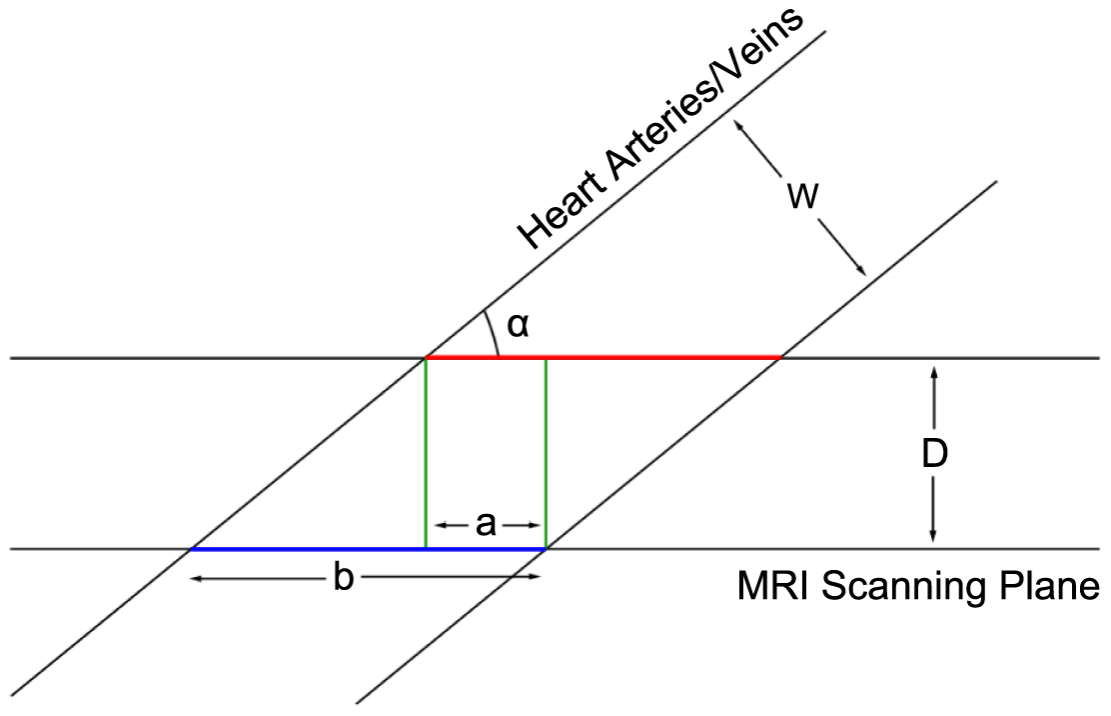
Determining Similarity Index Threshold. We present an idealized cross-sectional view of the aorta undergoing scanning in an MRI machine. There are two pairs of parallel lines, the horizontal pair signify a pair of adjacent MRI scanning planes, while the slanted pair of parallel lines is the cross-sectional view of an aorta that is being scanned. The ratio of *a* to *b* is the amount of 1-dimensional overlap that occurs when the red line is projected onto the blue line. The analogue of this overlap in 2-dimensions is a pair of intersecting ellipses.

For determining the minimum *similarity index* threshold value, we based our selection on a few basic assumptions within an idealized situation (see Figure 15). In the figure, the pair of horizontal lines represent a pair of adjacent MRI scanning planes, while another pair of inclined parallel lines represent the cross-sectional view of the aorta. The two pairs of parallel lines intersect at an angle of *a* degrees. The pair of MRI scanning planes has a separation distance of *D*, while the diameter of the scanned aorta is *w*. The reason for choosing the aorta is such that our input of short-axis aligned MRI images of the human heart is taken with respect to the left ventricle, and hence between the left atrium and the aorta, the orientation of the aorta is more likely to be more angled with respect to the left ventricle.

Our MRI inter-slice scanning distance ranges from 5mm to 10mm, while the diameter of a human aorta ranges from 25mm to 35mm, depending on the gender. A conservative assumption would therefore be that *w* is equal to 2.5 times of *D*. For *a*, we take the minimum possible angle that the aorta can likely make with the left ventricle to be at 15 degrees. In Figure 15, the 1-dimensional overlap between the two contours (shown in red and blue lines) is the ratio 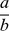. The value of 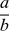 can be computed as
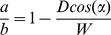
(11)


Using the value of 10mm for *D*, the resulting ratio is 0.61. Taking the computation to one higher dimension in order to compute the *similarity index*, it would be akin to measuring the perimeter intersection between two ellipses with an equation of 
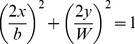
 and 
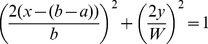
. Assuming that the two ellipses have an intersecting ratio of 0.61, their computed *similarity index* based on perimeter intersection would be 0.308. This is the underlying principle in using 30% as our minimum *similarity index* threshold.
